# Coding circular RNA in human cancer

**DOI:** 10.1016/j.gendis.2024.101347

**Published:** 2024-06-14

**Authors:** Yuan Lin, Yawen Wang, Lixin Li, Kai Zhang

**Affiliations:** Department of Breast Surgery, General Surgery, Qilu Hospital of Shandong University, Jinan, Shandong 250000, China

**Keywords:** Cancer, Cap-independent, Circular RNA, Protein-coding circRNA, Translation

## Abstract

circular RNA (circRNA) is a covalently closed single-stranded RNA that lacks 5' and 3' ends and has long been considered a noncoding RNA. With the development of high-throughput sequencing and bioinformatics technology, the understanding of circRNA has become increasingly advanced. Recent studies have shown that some cytoplasmic circRNAs can be effectively translated into detectable proteins, further indicating the importance of circRNA in cellular pathology and physiological functions. Internal ribosome entry site (IRES) and N^6^-methyladenosine (m^6^A) mediated cap-independent translation initiation are considered potential mechanisms of circRNA translation. Multiple circRNAs have been shown to play crucial roles in human cancer. This paper provides an overview of the nature and functions of circRNA and describes the possible mechanisms underlying the initiation of circRNA translation. We summarized the emerging functions of circRNA-encoded proteins in human cancer. Finally, we discuss the therapeutic potential of circRNAs and the challenges of research in this field. This review on circRNA translation will reveal a hidden human proteome and enhance our understanding of the importance of circRNAs in human malignant tumors.

## Introduction

circular RNA (circRNA) is produced by reverse splicing or nonlinear splicing reactions of precursor mRNA in mammalian nuclear and mitochondrial genomes. In recent years, circRNAs have attracted widespread attention in the field of tumor occurrence and development. In 1976, Sanger and his collaborators discovered circRNAs in viroids using electron microscopy.^1^ circRNAs were once considered nonbiological byproducts of abnormal splicing of exons.^2^ With the development of high-throughput sequencing technology and bioinformatics analysis, the study of circRNAs has made great progress, and many circRNAs have been identified in different species.^3^^,^^4^ circRNAs are usually divided into three types: intron circRNAs (CiRNAs), exon circRNAs (EcircRNAs), and exon–intron circRNAs (EIciRNAs). circRNAs are a unique class of noncoding RNAs, characterized by the absence of covalently closed loops at the 5' and 3' ends, and resistance to digestion by ribonucleases (such as RNase R).^5^^,^^6^ This makes them more structurally stable, more resistant to RNA exonucleases, and with a longer half-life of up to 10-fold greater than linear RNA.^7^ These circRNAs have stable structures, conserved sequences, and cell- or tissue-specific expression patterns.^8^

circRNAs regulate gene expression at the transcriptional and posttranscriptional levels, modulate cell proliferation, differentiation, and apoptosis, mediate cellular immune responses, and play important roles in the occurrence, progression, drug resistance, and metastasis of many cancers.^9^ circRNAs contain a large number of miRNA binding sites, and the inhibition of their target genes by miRNAs can be relieved by the sponging action of miRNA molecules. For example, hsa_circ_0025202 acts as an oncogenic circRNA in human epidermal growth factor receptor 2 (HER2)-positive breast cancer by regulating the miR_182_5p/FOXO3a (forkhead box O3) axis and increasing the sensitivity of breast cancer to tamoxifen treatment.^10^ In addition, circRNAs located in the nucleus may regulate the transcription of host genes through enhanced binding to RNA polymerase II.^11^ circRNAs may also interact with RNA binding proteins and regulate their biological function. Up-regulated circ_CYP24A1 can accelerate the malignant progression of esophageal squamous carcinoma by binding to pyruvate kinase M2 (PKM2) to activate the NF-κB (nuclear factor kappa B) pathway and promote the secretion of the tumor cell chemokine CCL5 (C–C motif chemokine ligand 5).^12^ Protein scaffold circRNAs can also serve as scaffolds to promote contact between two or more proteins. circ_Foxo3 acts as a protein scaffold that binds to p53 and the E3 ubiquitin-protein ligase Mdm2 (mouse double minute 2), facilitating Mdm2-induced ubiquitination, which in turn promotes p53 degradation.^13^ circRNAs can also recruit specific proteins, not only increasing the level of the protein but also promoting its nuclear translocation. For example, circ_Amotl1 not only increased the protein levels of signal transducer and activator of transcription 3 (STAT3) and DNA methyltransferase 3 alpha (Dnmt3a) but also recruited STAT3 from the cytoplasm to the nucleus, facilitated the nuclear translocation of STAT3 and stabilized its binding to the Dnmt3a promoter.^14^

Due to their lack of typical mRNA characteristics, circRNAs were previously considered as noncoding RNAs. With the development of high-throughput sequencing technologies, many translated circRNAs have been detected through RNA sequencing (RNA-seq) combined with polymer analysis and circRNA-specific bioinformatics algorithms.^15^, ^16^, ^17^, ^18^ Growing evidence suggests that some circRNAs can encode functional polypeptides through cap-independent translation mechanisms. Both internal ribosome entry site (IRES) and N^6^-methyladenosine (m^6^A) modifications promote the effective initiation of circRNA protein translation in human cells.^19^ circRNAs encode proteins involved in regulating a variety of pathological and physiological processes associated with cancer development and progression. In this review, we described the main mechanisms driving circRNA translation. In addition, we summarized the roles of circRNA-encoded proteins in tumorigenesis and progression. Finally, we discuss the therapeutic potential of translated circRNAs and the challenges in this field.

## Translation mechanism of circRNA

**Figure 1:**
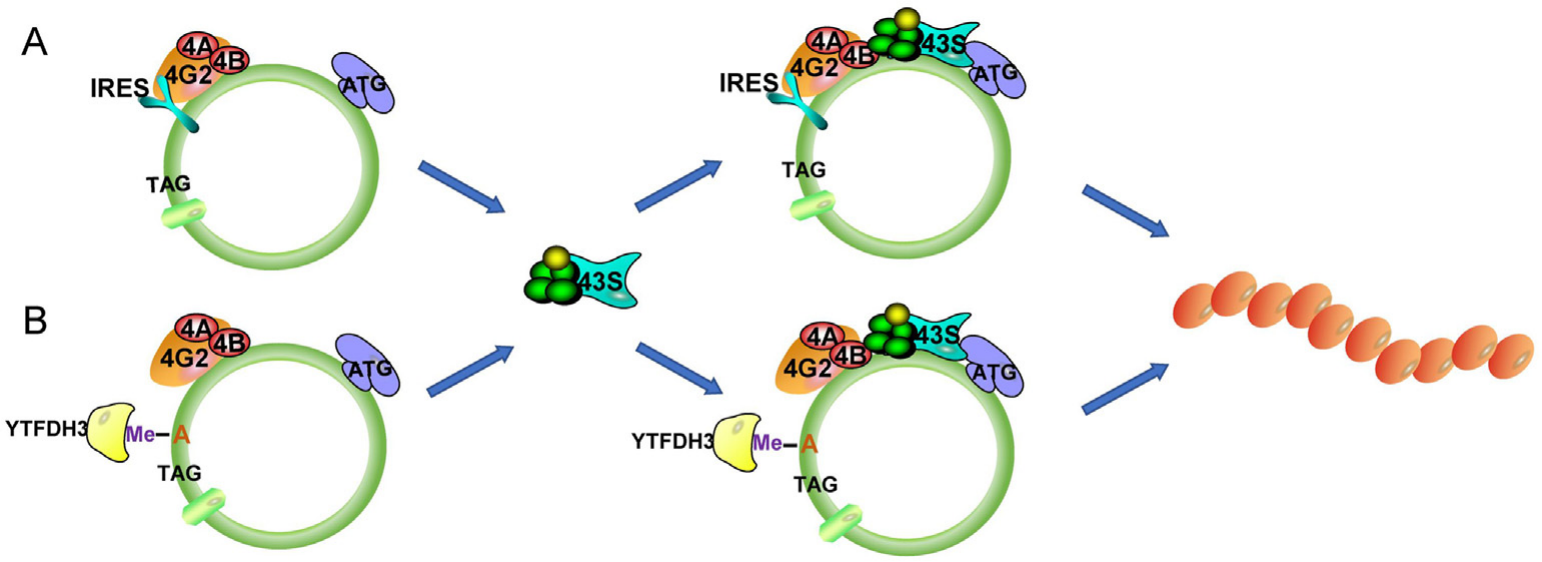
circRNA translation mechanism. **(A)** IRES initiates the translation of circRNAs. The atypical eIF4G protein (eIF4G2) directly recognizes the IRES on circRNA, assembles the eIF4 complex, and translates the downstream ORF. **(B)** MIRES initiates the translation of circRNAs. The m^6^A motif in circRNA is recognized by the m^6^A reader YTHDF3 and activated by the YTHDF3-eIF4G2 complex for subsequent translation. IRES, internal ribosome entry site; eIF4G2, eukaryotic initiation factor 4 gamma 2; eIF4, eukaryotic initiation factor; ORF, open reading frame; MIRES, m^6^A induced ribosome entry sites; m^6^A, N^6^-methyladenosine; YTHDF3, YTH N6-methyladenosine RNA binding protein F3.

According to classical RNA theory, eukaryotic mRNA requires a 5'-end m^7^-methylguanine nucleoside (m^7^G) cap and a 3'-end poly-A tail for protein synthesis in ribosomes. The translation of mRNA is cap-dependent and initiated by the cap-binding protein complex (eukaryotic initiation factor 4F complex, eIF4F), which consists of eIFs, eIF4E, eIF4G, and eIF4A. eIF4E binds to the 5' cap of mRNA, and eIF4G serves as the initiation complex for the assembly of the protein binding scaffold. eIF4A is a DEAD-box RNA-dependent ATPase that can unwind RNA duplexes with the help of eIF4B. It can also act as an RNA helicase to unlock secondary and tertiary structural regions at the 5' end, facilitating ribosome scanning. After binding eIF3 to eIF4G, the 43S preinitiation complex composed of eIFs, Met-tRNA_i_^Met^, and 40S subunits is recruited to mRNA to initiate translation.^20^, ^21^, ^22^ The greatest difference between circRNA and mRNA is that circRNA does not have a 5' m^7^G cap or a 3' poly-A tail. The translation of circRNAs is cap-independent, with initiation factors binding to the IRES or m^6^A to promote the formation of initiation complexes (Fig. 1).

### IRES-mediated translation

Most circRNAs contain natural IRESs that can be directly identified and regulated by nonclassical eIF4G proteins (eIF4G2 or DAP-5/death associated protein 5) to initiate circRNA translation. In contrast to eIF4G, eIF4G2 contains eIF4A and eIF3 binding regions but lacks eIF4E binding sites.^23^, ^24^, ^25^ Therefore, in IRES-driven translation, IRES can assemble eIF4 complexes in the absence of eIF4E and initiate translation directly.^26^ Chen et al revealed that artificially constructed emerging circRNAs containing IRES-like elements could recruit 40S ribosomal subunits, initiate translation, and produce long repetitive polypeptide chains with continuous open reading frames (ORFs).^27^ Another study further confirmed that circRNAs can effectively encode functional proteins by inserting IRES-like elements into circRNA reporter genes containing split green fluorescent protein (GFP).^28^ The above research shows that circRNAs can be translated into messenger RNAs that are translated into proteins through IRES-like elements independent of the 5' cap and 3' poly-A tail (Fig. 1A).

### m^6^A mediated translation

m^6^A is formed by methylation of adenosine residues in RNA. The cap-independent translation induced by m^6^A modification mainly occurs in the 5' untranslated region of certain circRNAs (Fig. 1B). circRNAs carry a putative ORF spanning the section of the m^6^A motif “RRACH” (R = G or A; H = A, C or U) in the 5' untranslated region. Yang et al inserted a short fragment (19 nt) containing a copy of the consensus m^6^A motif before the start codon of the circRNA reporter gene and measured the production of GFP in transfected cells. As expected, circRNAs containing one or two m^6^A motifs are effectively translated into GFPs, and mutations in these two motifs significantly reduce (but do not eliminate) GFP levels.^28^ m^6^A is specifically recognized by the YTH domain family proteins YTHDF1, YTHDF2, and YTHDF3, which bind to m^6^A and function as m^6^A readers. YTHDF3 can recognize m^6^A, and this complex combines with the initiation factor eIF4G2, which anchors and attaches to the 40S ribosome subunit complex and the 43S complex, inducing translation. Yang et al also showed that short sequences containing m^6^A sites can act as m^6^A-induced ribosome entry sites (MIRES) to promote cap-independent translation of circRNAs.^28^

## Techniques and methods for identifying circRNA coding ability

circRNAs with coding potential can be identified using bioinformatics methods. TransCirc is a multiomics circRNA translation database that integrates a variety of evidence related to circRNA translation, provides a visual representation of the relevant evidence for translation products, such as protein profiling, ribosome profiling, or polyribosome analysis, and integrates IRES sequence elements, circRNAs of m^6^A modification sites, translation initiation sites, and ORFs.^29^ RiboCirc is a data-oriented translation database that combines a wide range of translation-related information and contains 3168 existing Ribo-seq and 1970 paired RNA-seq, covering 314 studies and 21 species. Finally, more than two thousand circRNAs with translational potential were identified via Ribo-seq/RNA-seq pairing.^30^ CircRNADb is a comprehensive circRNA information query database that annotates the IRES sequence elements and ORFs of circRNAs with protein-coding potential and provides mass spectrometric evidence for protein expression. In addition, the database shows the properties of circRNAs, including domains, N-glycosylation sites, mucin O-glycosylation sites, and phosphorylation sites.^31^ The CircAtlas database provides detailed information about circRNAs by identifying IRESs and ORFs in full-length circRNAs to predict their coding potential.^32^ The CircBank database provides circRNA m^6^A modification data and IRES site information to predict circRNA coding ability.^33^ The IRESite database is based on experimental data for 68 viruses and 115 eukaryotic cells and contains a large number of genes with IRES sites. The sequences of circRNAs of interest can be compared to predict whether circRNAs have IRES sites.^34^ In addition, CRAFT is a bioinformatics software that can predict the coding potential of a putative ORF to a circRNA using the ORF finder tool.^35^ CircPrimer 2.0 is a Java-based software that annotates circRNAs and predicts the ORF, IRES, and m^6^A loci of circRNAs.^36^

The identification of circRNA-encoding functions has focused mainly on sequence translation ability verification, endogenous peptide detection, translation regulatory element detection, and peptide function studies. Methods commonly used to identify and validate circRNA encoding capabilities include the following: i) polyribosome profiling and ribosome profiling (Ribo-seq), which are transcriptomic methods used to identify unknown endogenous translatable circRNAs; ii) the translation ability of the sequence was verified by dual luciferase vector system and flag-tagged protein expression followed by Western blot detection; iii) endogenous proteins were detected by specific antibodies and liquid chromatography-tandem mass spectrometry; iv) m^6^A-RIP-seq detects m^6^A modifications around the start codon; v) ribosome nascent-chain complex-bound RNA sequencing (RNC-Seq) can detect the translation of circRNAs associated with ribosomes, which are enriched and analyzed by RNA-seq to detect ribosome binding locations, and then infer the start codon location, translation pause, termination location, and true ORF; vi) the specific peptides encoded by endogenous circRNAs were verified by mass spectrometry; vii) dual luciferase vectors were used to detect the activity of IRES-like elements on circRNAs.

## circRNAs encoded proteins in human malignant tumors

Recent evidence suggests that aberrant expression of circRNAs occurs in almost all malignant tumor types and that circRNAs play a crucial role in tumor pathogenesis by acting as suppressors and oncogenes.^37^ The protein products encoded by circRNAs exert important biological functions through different mechanisms in the occurrence and development of various malignant tumors (Table 1).

**Table 1:**
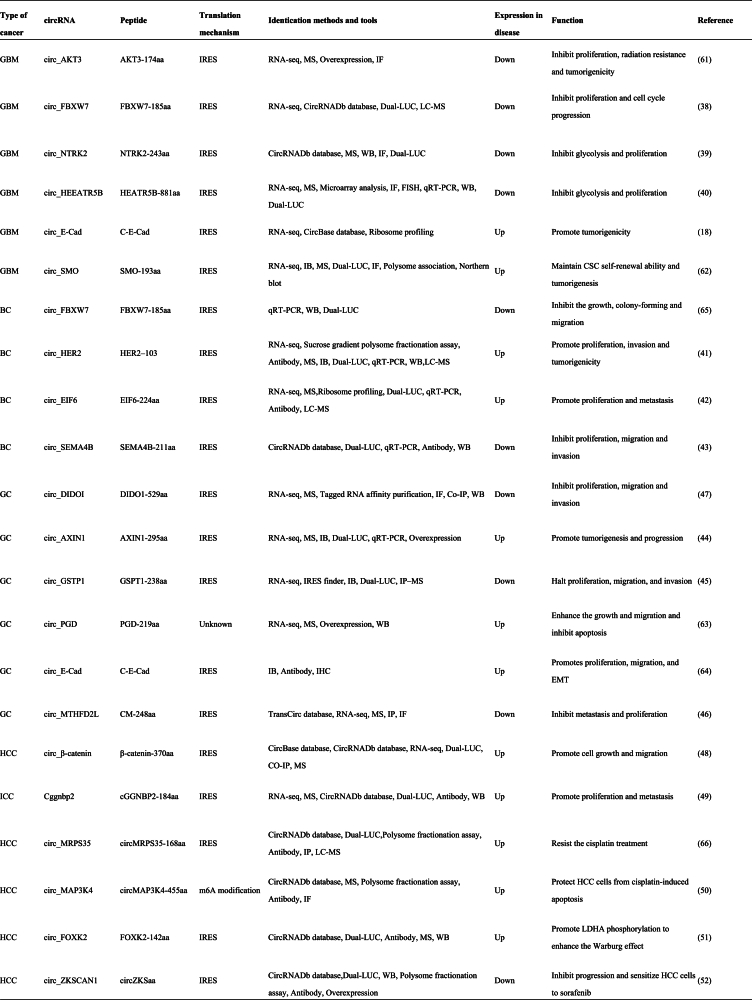

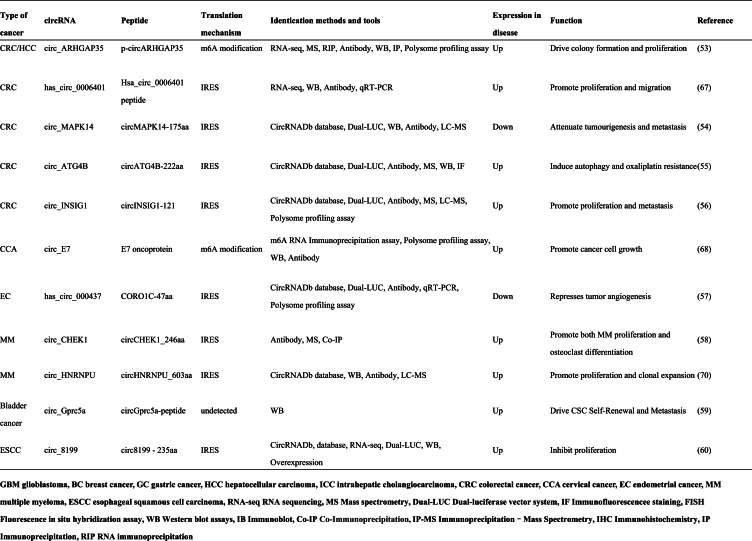
Proteins encoded by tumor related CircRNA and their functions.

### circRNA-encoded peptides/proteins interact with proteins

#### Glioblastoma (GBM)

circ_FBXW7 driven by an IRES, encodes a novel 21-kDa protein in GBM, termed FBXW7-185aa.^38^ circ_FBXW7 expression is positively associated with overall survival in GBM patients, while FBXW7-185aa induces cell cycle arrest and reduces proliferation by competitively interacting with USP28 (ubiquitin specific peptidase 28) and releasing FBXW7α (F-box and WD repeat domain containing 7 alpha) to degrade c-Myc in glioma cells. In GBM, muscle blind splicing regulatory factor 1 (MBNL1) promotes the expression of circ_NTRK2 by binding to neurotrophic receptor tyrosine kinase 2 (NTRK2) precursor mRNA. circ_NTRK2 encodes a new protein, NTRK2-243aa, which phosphorylates paired box 5 (PAX5) at Y102, resulting in a shortened PAX5 half-life and inhibition of glycolysis in GBM cells.^39^ Gao et al reported an undescribed secretory variant of the E-cadherin protein (C-E-Cad) encoded by circ-E-Cad RNA. The unique 14 amino acids at the C-terminus of C-E-Cad bind to the EGFR CR2 domain, activating epidermal growth factor receptor (EGFR) and maintaining the tumorigenicity of glioma stem cells.^18^ circ_HEATR5B encodes a new protein HEATR5B-881aa, which directly interacts with Jumonji C-domain containing 5 (JMJD5) and reduces the stability of JMJD5 by phosphorylating S361, thereby inhibiting glycolysis and proliferation in GBM cells.^40^

#### Breast cancer (BC)

HER2-103 is a novel protein encoded by circ_HER2 and shares an amino acid sequence that is partially identical to the HER2 CR1 structural domain.^41^ HER2-103 can interact with EGFR/HER3 to promote homologous/heterologous dimerization of EGFR/HER3, maintaining AKT (protein kinase B) phosphorylation and the downstream malignant phenotype. Pertuzumab significantly attenuated *in vivo* tumorigenicity in triple-negative breast cancer (TNBC) cells expressing circ-HER2/HER2–103, but did not affect tumorigenicity in circ-HER2/HER2–103 negative TNBC cells. circ-EIF6 is translated into EIF6-224aa in an IRES-dependent manner, and EIF6-224aa is endogenously expressed in TNBC cell lines and tissues. Direct interaction between EIF6-224aa and the oncogene MYH9 in breast cancer inhibits the ubiquitin-proteasome pathway mediated activation of the Wnt/β-catenin pathway, thereby reducing the degradation of MYH9.^42^ circ_SEMA4B and the newly encoded protein SEMA4B-211aa were significantly down-regulated in BC tissues and exerted tumor suppressive effects *in vivo* and *in vitro*^43^ SEMA4B-211aa inhibits AKT (Thr308) phosphorylation by inhibiting PIP3 (phosphatidylinositol-3,4,5-trisphosphate) production through competition with p110 and binding to p85. circ_SEMA4B also inhibits AKT (Ser473) phosphorylation through the miR330-3p/PDCD4 (programmed cell death factor 4) axis.

#### Gastric cancer (GC)

Peng et al identified a new functional protein, AXIN1-295aa, encoded by circ_AXIN1, which is highly expressed in GC.^44^ AXIN1-295aa acts as a carcinogenic protein and competitively interacts with the tumor suppressor gene APC (adenomatous polyposis coli), resulting in disruption of the “destruction complex” of the Wnt pathway. Subsequently, by transactivating the classical Wnt pathway and inducing the expression of Wnt-dependent genes, β-catenin accumulates in the nucleus to promote cell proliferation and migration. Hu et al reported that circ_GSPT1 was expressed at low levels in GC. circ_GSTP1 driven by IRES encodes a functional peptide named GSPT1-238aa. This peptide exerts an anti-tumor effect *in vitro* independent of host genes. The interaction between the vimentin/Breclin1/14-3-3 complex and GSPT1-238aa ultimately regulates autophagy in GC cells.^45^ Lui et al identified the protein CM-248aa encoded by circ_MTHFD2L.^46^ CM-248aa was significantly down-regulated in GC, and its low expression was associated with poorer TNM staging and histopathological grading. CM-248aa rescued the activity of protein phosphatase 2A (PP2A) by competitively binding to SET. CM-248aa down-regulated SET and inhibited PP2A activity, resulting in downstream pathways and GC progression. circ_DIDO1 has powerful tumor inhibitory activity in GC. A novel 529aa protein encoded by circ_DIDO1 can inhibit the growth and invasion of GC cells. Direct interaction between this protein and poly ADP-ribose polymerase 1 (PARP1) DNA binding domain inhibits the DNA repair ability of GC cells and causes PRDX2 (peroxiredoxin 2) ubiquitination and degradation.^47^

#### Liver cancer

Liang et al revealed that knocking down circβ-catenin inhibited the Wnt/β-catenin pathway and demonstrated that circβ-catenin could produce a novel β-catenin isoform containing 370-amino acids termed β-catenin-370aa.^48^ This novel β-catenin isoform promotes hepatocellular carcinoma (HCC) proliferation and metastasis by antagonizing GSK3β (glycogen synthase kinase 3 beta)-induced β-catenin phosphorylation and degradation, stabilizing full-length β-catenin, and activating the Wnt pathway. The circRNA cGGNBP2 in IL-6-stimulated intrahepatic cholangiocarcinoma cells in which the encoded protein cGGNBP2-184aa was driven by an IRES.^49^ The direct interaction between CGGNBP2-184aa and STAT3 promotes STAT3Tyr705 phosphorylation and plays a positive regulatory role in regulating IL-6/STAT3 signaling, promoting intrahepatic cholangiocarcinoma cell proliferation and metastasis. Driven by m^6^A modification, circ_MAP3K4 encodes the protein circMAP3K4-455aa.^50^ circMAP3K4-455aa interacts with AIFM1 (apoptosis-inducing factor mitochondria associated 1), and prevents cisplatin-induced apoptosis in HCC cells. Recently, Zheng et al have shown that circ_FOXK2 enhances the Warburg effect in HCC cells by up-regulating the expression of Fis1 (mitochondrial fission protein 1) and inducing mitochondrial division through sponging miR-484. circ_FOXK2 contains a 429-nt ORF encoding a protein FOXK2-142aa containing 142 amino acids. The interaction between FOXK2-142aa and the C-terminal subunit of LDHA (lactate dehydrogenase A) activates phosphorylation of LDHA at the residue Tyr10, which enhances the Warburg effect and facilitates the progression of HCC.^51^ circ_ZKSCAN1 encodes a polypeptide containing 206 amino acids, named circZKSaa, which is up-regulated in sorafenib-treated HCC cells. This may sensitize HCC cells to sorafenib by promoting mTOR (target of rapamycin) ubiquitination via interacting with FBXW7, inhibiting the classical PI3K (phosphoinositide 3-kinase)/AKT/mTOR pathway in HCC pathogenesis, and ultimately inhibiting HCC cell proliferation.^52^

#### Colorectal cancer (CRC)

circ_ARHGAP35 encodes an oncogenic protein driven by m^6^A, which promotes colony formation and proliferation in cancer lines by interacting with the TFII-I (transcription factor II-I) protein in the nucleus in CRC.^53^ Its homologous linear mRNA is a tumor suppressor that inhibits cell migration and invasion. However, a novel protein, circMAPK14-175aa, is encoded by circ_MAPK14 via an IRES and is expressed in CRC tissues and cells.^54^ circMAPK14-175aa prevents metastasis and progression by promoting ubiquitin-mediated FOXC1 (forkhead box C1) degradation by competitively binding to the upstream kinase MKK6. circ_ATG4B encodes a novel protein, circATG4B-222aa, which can act as a decoy to interact competitively with TMED10 (transmembrane P24 trafficking protein 10) and inhibit the binding of TMED10 to ATG4B (autophagy-related gene 4B), thereby increasing autophagy and inducing the development of chemotherapy resistance.^55^ circINSIG1 encodes a novel protein, circINSIG1-121, which interacts with the CUL5-ASB6 complex and promotes the ubiquitination of the key cholesterol metabolism regulator INSIG1 (insulin induced gene 1) at lysine residues 156 and 158 of K48, thereby facilitating cholesterol biosynthesis and contributing to CRC proliferation and metastasis.^56^

#### Other cancers

Hsa_circ_0000437 encodes the functional peptide CORO1C-47aa through a short ORF.^57^ CORO1C-47aa competes with the transcription factor TACC3 (transforming acidic coiled-coil containing protein 3) for binding to ARNT (aryl hydrocarbon receptor nuclear translocator) via the PAS-B structural domain, resulting in transcriptional inhibition of VEGF (vascular endothelial growth factor) and ultimately reducing endometrial cancer angiogenesis. The peptide circCHEK1-246aa is expressed in multiple myeloma (MM) cells and is encoded by the circRNA circCHEK1 (hsa_circ_0024792). Mature circCHEK1-246aa is secreted into the bone marrow microenvironment, interacts with natural centrosome protein 170 (CEP170), and affects the expression of mutant CEP170 in MM cells. circCHEK1-246aa exacerbates MM by inducing chromosomal instability and the formation of bone lesions.^58^ circ_Gprc5a functions in a peptide-dependent manner and has peptide coding potential. Gprc5a is a surface protein that is highly expressed on bladder stem cells to which the circ_Gprc5 peptide binds. Targeted therapy for bladder cancer and bladder cancer stem cells can be achieved via the circ_Gprc5a-peptide-Gprc5a axis.^59^ The 235aa polypeptide encoded by circ_8199 is mainly located in the cytoplasm, and can inhibit esophageal squamous-cell carcinoma cell proliferation by binding to and inhibiting the activity of OGT (O-linked N-acetylglucosamine transferase) through the JAK2 (Janus kinase 2)-STAT3 pathway.^60^

### circRNA-encoded peptides/proteins regulate signaling pathways

circ_AKT3 encodes a protein of 174 amino acids named AKT3-174aa. AKT3-174aa competes with phosphorylated PDK1 (pyruvate dehydrogenase kinase 1), blocks AKT-thr308 phosphorylation, regulates PI3K/AKT signaling, and inhibits GBM cell proliferation, radiation resistance, and anti-tumor activity *in vivo*.^61^ In addition, Wu et al revealed that circ_SMO encodes a new oncogenic protein, SMO-193aa, which is driven by an IRES and enhances the cholesterol modification of SMO (smoothened, frizzled class receptor) by interacting with SMO, and releases SMO from patched transmembrane receptor inhibition.^62^ SMO-193aa in brain cancer stem cells attenuated Hedgehog signaling and suppressed their self-renewal and proliferation *in vitro* and tumorigenesis *in vivo*.

circ_PGD encodes an oncogenic protein, PGD219aa containing a reverse splicing site, which promotes the growth and migration of GC cells and inhibits GC cell apoptosis through the SMAD2/3 (SMAD family member 2/3) and YAP signaling pathways. PGD-219aa can also affect apoptosis, epithelial–mesenchymal transition, and signaling pathway related proteins in GC cells.^63^ circ_E-Cad encodes a 254-amino-acid protein, C-E-Cad, which promotes glioblastoma progression through the PI3K/Akt pathway.^18^ Recently, Li et al reported that both circ-E-Cad and C-E-Cad expression were up-regulated in GC cell lines and tissues compared with normal tissues. This finding suggested that circ_E-Cad may be a promising GC biomarker. In addition, their studies revealed for the first time that protein C-E-Cad was highly expressed in GC, and that the TGF-β (transforming growth factor-beta)/Smad pathway increased the expression of C-ECad, which promoted tumorigenesis and invasion by affecting PI3K/AKT signaling to regulate the proliferation, migration, and epithelial–mesenchymal transition of GC cells.^64^

### Others

As mentioned above, circ_FBXW7 is reported to encode a novel protein in GBM with anti-tumor effects. In addition, Ye et al revealed that the FBXW7-185aa protein encoded by circ_FBXW7 can increase the abundance of FBXW7 (F-box and WD repeat domain containing 7) and induce c-Myc degradation, thus inhibiting the migration and proliferation of TNBC cells.^65^ circ_MRPS35 acted as a miRNA sponge to form the circ_MRPS35-miR148a-STX3 (syntaxin 3)-PTEN (phosphatase and tensin homologue deleted on chromosome 10) axis, which promoted the malignant progression of HCC. In addition, circ_MRPS35 encodes a 168-amino-acid polypeptide named circMRPS35-168aa.^66^ The chemotherapeutic drug cisplatin can significantly induce circMRPS35-168aa, which results in cisplatin resistance in HCC cells by inhibiting cisplatin-induced cell apoptosis. The circRNA hsa_circ_0006401 is derived from col6a3 (collagen type VI alpha 3 chain), which contains an ORF that encodes a novel functional peptide of 198 amino acids.^67^ The hsa_circ_0006401 peptide promotes col6a3 mRNA stability, which promotes CRC cell proliferation and metastasis. The E7 oncoprotein is a product of the translation of circE7 from human papillomavirus 16 and contains 98 amino acids. The ability of the E7 tumor protein to inhibit the growth of cervical cancer cells suggests that the translation of viral-derived circRNAs may be associated with the transforming properties of certain human papillomaviruses.^68^ circ_HNRNPU is secreted by MM cells and encodes a circHNRNPU-603aa protein. circHNRNPU_603aa includes the RNA-binding RGG-box region that regulates the SKP2 exon jump, stabilizes c-Myc in MM by competitively inhibiting c-Myc ubiquitination, and promotes MM cell proliferation.^69^

## Translatable circRNAs as therapeutics

**Figure 2:**
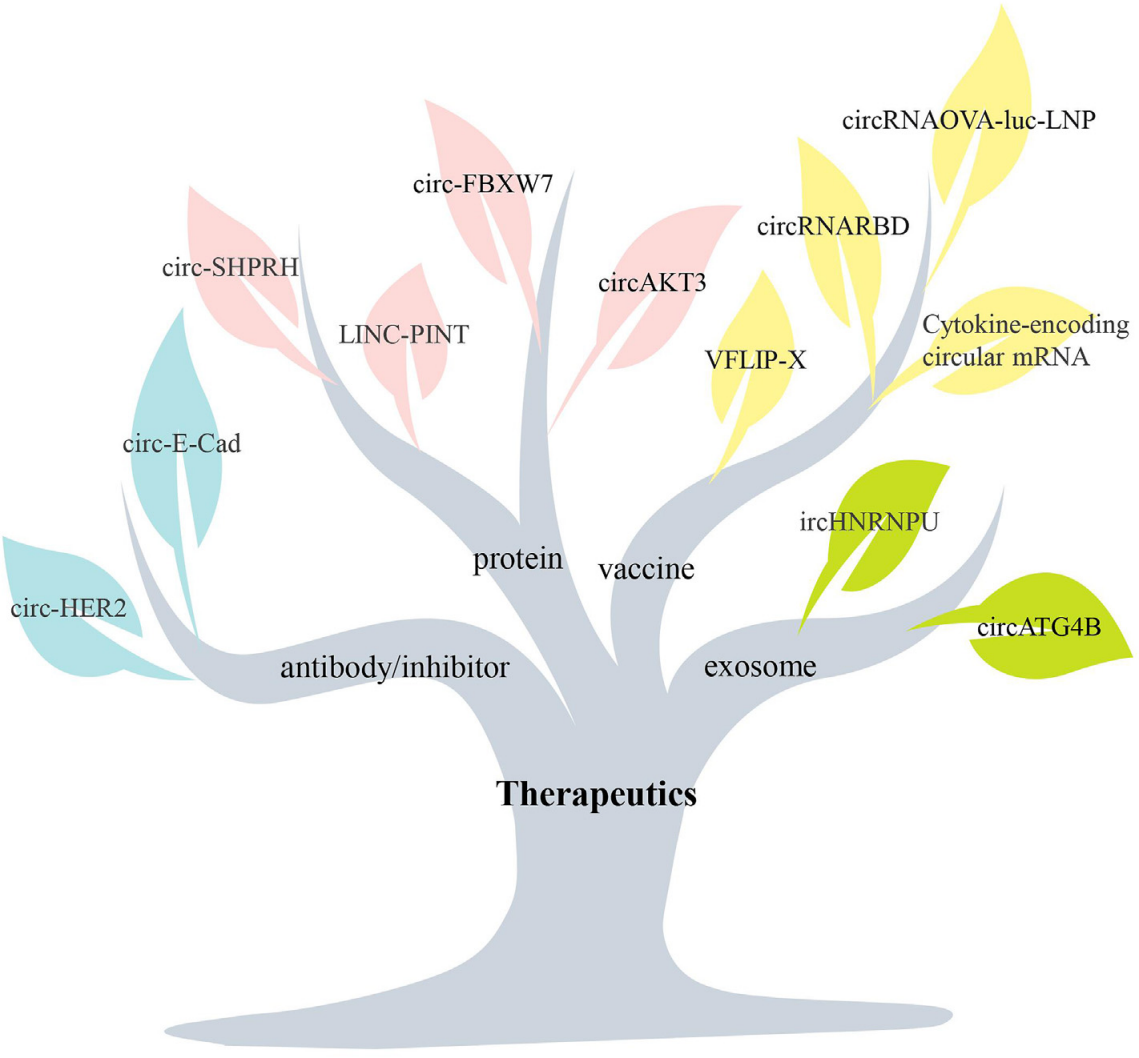
Translatable circRNAs as therapeutics.

Many studies have shown that circRNA-encoded proteins/peptides play a role in tumor metabolism, signaling pathways, cell proliferation, angiogenesis, metastasis, and other processes, and exert anti-tumor or oncogenic effects through different signal transduction pathways.^9^ With further research, there may be ways that circRNAs and their encoded proteins can be applied to the treatment of tumors via various routes (Fig. 2).

### Protein replacement therapy

New targeted therapeutic agents based on these novel peptides/proteins that indirectly inhibit cancer cell growth by targeting tumor pathways, inducing an immune response, or directly inducing cancer cell apoptosis by recruiting specific pathways have been developed.^70^^,^^71^ For example, the CIRC-FBXXW7-185aa protein encoded by circ-FBXW7 can prevent malignant glioma cell proliferation and cell cycle acceleration by down-regulating c-Myc protein expression and blocking the progression and metastasis of TNBC. Therefore, circFBXW7 can be used as a therapeutic target and prognostic biomarker for TNBC. AKT3-174aa, encoded by circAKT3, can act as a tumor suppressor. AKT3-174aa inhibits malignant phenotypes caused by AKT activation. By participating in the RTK (receptor tyrosine kinase)/PI3K/AKT signaling pathway, AKT3174aa regulates glioma development. Natural circRNA encodes an 87-amino-acid peptide called PINT87aa, which inhibits glioblastoma tumorigenesis.^72^ circ-SHPRH is a natural circRNA that encodes a protein called SHPRH-146aa, which is involved in the onset of neurodegenerative diseases.^73^ circZKSCAN1 encodes circZKSaa, which plays a tumor-inhibiting role in the PI3K/AKT/mTOR pathway and sensitizes HCC cells to the first-line drug sorafenib by mediating the ubiquitination of mTOR to inhibit HCC development.^52^

### Vaccines

Cancer vaccines, which induce anti-tumor responses by expressing antigens in the body, have received much attention in recent years. With the development of biotechnology and molecular medicine, artificial circRNAs have been designed as a new type of vaccine for disease treatment and prevention.^74^^,^^75^ The application of linear mRNA vaccines is limited by their instability, inefficiency, and innate immunogenicity. In contrast, circRNA vaccines containing IRES and ORF are safer, more stable, simpler to manufacture, and more scalable. In addition to being translated into proteins for disease prevention and treatment, engineered circRNAs are also used to express related antigens to trigger adaptive immune responses and disease treatment. For example, Wei et al synthesized a circRNA vaccine, circRNARBD, in which the binding domain (RBD) encodes the SARS-CoV-2 spike protein to inhibit the SARS-CoV-2 virus. A SARS-CoV-2 circRNA vaccine encoding a relatively stable VFLIP-X spike protein has been identified for use as a next-generation COVID-19 vaccine against existing and developing SARS-CoV-2 variants. The potential of circRNAs as anti-cancer vaccines has been demonstrated for the treatment of melanoma in mice.^76^ A circRNA-LNP platform was designed to study the function of circRNA vaccines *in vivo*. The OVA (257–264)-luciferase-coding circRNA (circRNA^OVA-luc^) was purified and encapsulated in lipid nanoparticles to form a stable complex. The circRNA-LNP vaccine activated a strong innate immune response and an effective antigen-specific T-cell response in the body, demonstrating remarkable anti-tumor effects in mouse tumor models. Recently, a new circular mRNA named cmRNA, as a novel circRNA vaccine, was shown to significantly inhibit tumor growth in syngeneic mouse models of colon cancer and melanoma by encoding a mixture of cytokines (interleukin-15, interleukin-12, granulocyte-macrophage colony-stimulating factor, and interferon-alpha 2b). In addition, it promotes anti-PD-1-mediated immunotherapy.^77^

### Antibodies/inhibitors

Over the past 30 years, therapeutic antibodies have revolutionized the field of targeted cancer therapy. At present, therapeutic antibodies have been rapidly developed. Due to the precise targeting ability of antibodies, they attack and kill tumor cells through various mechanisms and have become an important part of modern biomedicine.^78^ For translatable circRNA, in terms of therapeutic potential, not only circRNA itself but also the protein it encodes can be targeted, and its function can be inhibited by accurately inhibiting the activity of the encoded protein. circ-E-Cad RNA encodes the 254-amino-acid protein C-E-Cad, which binds to the EGFR CR2 domain through its unique 14-amino-acid carboxyl terminus and activates EGFR, thereby maintaining the tumorigenicity of glioma stem cells. Notably, one study blocked EGFR signaling by targeting C-E-Cad, enhancing anti-EGFR therapy, and inhibiting the tumorigenicity of glioblastoma stem cells.^18^ The CIRC-HER2-encoded protein HER2-103 shares most of the same amino acid sequence as the HER2 CR1 domain and can be antagonized by the anti-HER2 monoclonal antibody drug pertuzumab. Pertuzumab significantly reduced the tumorigenic capacity of TNBC cells expressing circ-HER2/HER2-103.^41^

### Exosomal circRNA

Previous studies have shown that human serum exosomes contain a large number of intact and stable circRNAs. Exosomal circRNAs can be delivered to recipient cells and induce functional responses and phenotypic changes such as tumor immunity, tumor progression and metastasis, angiogenesis, drug resistance, and tumor metabolism in cancer.^79^ circHNRNPU is secreted by MM cells and encodes a protein called circHNRNPU_603aa. circHNRNPU_603aa overexpression promoted the proliferation of MM cells. These effects were eliminated by siRNA-mediated knockout of circHNRNPU_603aa.^69^ Exosome circATG4B was found to encode a new protein CircatG4B-222AA in CRC, and CircatG4B-222AA interacts with TMED10, preventing TMED10 from binding to ATG4B, thereby leading to increased autophagy and inducing oxaliplatin resistance in CRC cells.^55^ Based on the stability and targeting specificity of exosomes to tissues or cells, exosomes are considered good delivery tools for circRNA-targeting agents and circRNA expression vectors. Exosomal circRNAs derived from oncogenic proteins can inhibit tumor progression by inhibiting their delivery. Promoting the delivery of some exosome circRNA-derived anti-tumor proteins can effectively inhibit the occurrence and progression of tumors.

circRNAs have great potential for development and application in the treatment of tumors. At present, many circRNAs have been found to be localized in the cytoplasm, and understanding the pathway by which they are transported from the nucleus is an important step in using circRNAs for therapeutic purposes, laying the foundation for the next generation of RNA therapies, which may lead to more potent, longer lasting, and potentially more versatile circRNA drugs.^80^ In addition, Chang's team developed a modular circRNA assembly platform for maximum translation of circRNA, which increased circRNA protein production several hundredfold and enabled efficient and durable protein production *in vivo*.^81^ This provides strong support for the development of translatable circRNAs as therapeutics and vaccines.

The stability and unique conformation of circRNAs may make them better than their linear RNA counterparts, and proteins expressed by them have great potential in cancer therapy, but there are still some problems to be solved regarding the translation function of circRNAs. First, the precise regulatory mechanism of circRNA translation is still unclear, and whether there are other initiating sequences, mechanisms, and precise regulatory, extension, and termination processes involved in the circRNA translation process needs further research. Second, why do the proteins expressed from these circRNAs in the cytoplasm have different localizations and functions than the host proteins? In addition, the circRNA synthesis method has several limitations, such as low cyclization efficiency and high cost of enzymes and other reagents. In the future, it is necessary to develop and improve advanced methods for the design, synthesis, purification, delivery, and therapeutic application of artificial circRNA vaccines *in vitro*.

## Conclusions and future perspectives

Most of the reported circRNAs encode proteins through IRES drives, and m^6^A-driven circRNA translation has also been reported. In recent years, circRNA-encoded proteins have been shown to be involved in the regulation of human physiology and pathology and play anti-tumor or oncogenic roles through different signal transduction pathways, indicating the importance of circRNA-encoded proteins in the development of tumors. This opens up therapeutic applications for circRNAs, including cancer diagnosis and treatment. For example, these peptides/proteins can be used as new targeted drugs or combined with anti-cancer drugs to inhibit the development of tumors or as viral vector vaccines. Investigating the role of circRNA-encoded proteins in human disease will help us elucidate the function of these peptides/proteins and develop potential tools for early detection and effective cancer treatment.

## Author contributions

K.Z. conceived the idea. K.Z., Y.W., Y.L., and L.X. collected all the references and designed and drew all figures. L.X. made table. K.Z. and Y.W. supervised the whole work and revised the manuscript. Y.W. and Y.L. wrote the manuscript. All authors read and approved the final manuscript.

## Conflict of interests

The authors declared no competing interests.

## Funding

This work was supported by the National Natural Science Foundation of China (No. 81802406), Shandong Provincial Natural Science Foundation of China (No. ZR2021MH045, ZR2020LZL009, ZR2019BH061, ZR2021MH225), and Special Funds for Scientific Research on Breast Diseases of Shandong Medical Association of China (No. YXH2021ZX058).
